# Comparative Efficacy and Acceptability of Anti-inflammatory Agents on Major Depressive Disorder: A Network Meta-Analysis

**DOI:** 10.3389/fphar.2021.691200

**Published:** 2021-07-01

**Authors:** Xiaoyi Hang, Yijie Zhang, Jingjing Li, Zhenzhen Li, Yi Zhang, Xuanhao Ye, Qisheng Tang, Wenjun Sun

**Affiliations:** Third Affiliated Hospital, Beijing University of Chinese Medicine, Beijing, China

**Keywords:** major depressive disorder, anti-inflammatory, network meta-analysis, efficacy, acceptability

## Abstract

**Background:** With the growing importance of research about the association between neuroinflammation and major depressive disorder (MDD), anti-inflammatory agents have been used as a new antidepressant therapy in clinical practice. We conducted a network meta-analysis (NMA) with up-to-date evidence to compare different anti-inflammatory agents for improving the treatment of MDD patients.

**Methods:** To identify eligible randomized clinical trials, four databases (i.e, the Cochrane Library, Web of Science, PubMed and Embase) were searched from inception date to May 31, 2020. Anti-inflammatory agents were defined as non-steroidal anti-inflammatory drugs (NSAIDs), corticosteroids, cytokine inhibitors, statins, pioglitazone, minocycline, N-acetylcysteine (NAC) and omega-3 fatty acid (Omega-3 FA). The main outcomes of this NMA were efficacy, acceptability and remission rate. Risk ratio (RR) was adopted for dichotomous outcomes, and the confidence interval (CI) was set at 95%. STATA 14.0 and R 3.6.3 were used to conduct the NMA. The study protocol was registered with PROSPERO (CRD42020182531).

**Results:** A total of 39 studies, involving 2871 participants, were included in quantitative data synthesis. For efficacy, NSAIDs (RR=0.50, 95%CI: 0.26-0.73) and pioglitazone (RR=0.45, 95%CI: 0.20-0.84) were more favorable than placebo. With respect to acceptability, NSAIDs were more acceptable than placebo (RR=0.89, 95%CI: 0.77-0.99) and minocycline (RR=1.22, 95%CI: 1.03-1.49). For remission, NSAIDs were more superior than placebo (RR=0.48, 95%CI: 0.27-0.79) and Omega-3 FA (RR=2.01, 95%CI: 1.09-3.90), while NACs were more favorable than placebo (RR=0.39, 95%CI: 0.13-0.99). Based on the surface under the cumulative ranking curve (SUCRA) value, corticosteroids (0.86) were the best anti-inflammatory agent for MDD patients in terms of efficacy, but the head-to-head comparisons for the efficacy of glucocorticoids and other agents were not statistically significant. As for acceptability, NSAIDs (0.81) were much better than other anti-inflammatory agents. Besides, NAC (0.80) was the best anti-inflammatory agent in the terms of remission.

**Conclusions:** In summary, we found that corticosteroids were more superior than other agents in terms of efficacy according to the SUCRA value. However, this result must be interpreted with caution because the head-to-head comparisons for the efficacy of glucocorticoids and other agents did not reach statistical significance. NSAIDs were recommended for acceptability and NAC for remission rate.

## Introduction

As a common mental illness, major depressive disorder (MDD) has been estimated to bring more than US$210 billion economic burden in the United States alone, while affecting over 350 million people around the world (Ferrari Alize et al., 2013; World Health Organization, 2017). MDD is often characterized by depressed mood, anhedonia and loss of interest (Global Burden of Disease Study, 2013 Collaborators, 2015). Antidepressant agents are mainly prescribed to treat MDD patients (Cipriani et al., 2018). However, about 30% patients had no improvement or partial responses, accompanied by high relapse and recurrence rates (Rush et al., 2006; Al-Harbi, 2012). Moreover, these patients endured the side effects of antidepressants, including weight gain, insomnia, nausea, cardiovascular toxicity, metabolic disorders, and even suicidal ideation (Papakostas, 2008). Therefore, it is of utmost urgency to develop new treatments and novel therapeutic targets for this disease.

Nowadays, neuroinflammation has been recognized as a causal factor or contributing cause for the development of MDD (Köhler et al., 2016). The immune responses and inflammation within the central nervous system (CNS) are collectively labeled as neuroinflammation, and microglia is one of the most pivotal members of neuroinflammatory cells involved in these reactions. Upon activation, the majority of microglia become amoeboid and induce the production of pro-inflammatory cytokines (TNF-α, IL-1β and IL-6) in the CNS. These cytokines activate mitogen-activated protein kinase (MAPK) and subsequently increase the levels of presynaptic transporters, which in turn suppress the activities of dopamine, noradrenaline and serotonin in the presynaptic neuron synapses. Moreover, these cytokines regulate the activation of indoleamine 2,3 dioxygenase (IDO) enzyme that metabolizes tryptophan into kynurenine, thereby reducing the availability of this serotonin precursor. The activated microglia also promotes the conversion of kynurenine into quinolinic acid, leading to the increased production of glutamate. Excess accumulation of glutamate can inhibit the synthesis of brain-derived neurotrophic factor (BDNF), thus affecting neuronal integrity (Roman and Irwin, 2020). It has been reported that a significant proportion of MDD patients exhibit increased levels of TNF-α and IL-6 (Uher et al., 2014; Yoshimura et al., 2009; O'Brien et al., 2007; Motivala et al., 2005; Pike and Irwin, 2006). Besides, randomized clinical trials have been conducted to determine whether anti-inflammatory agents, either as monotherapy or adjunctive therapy, can exert antidepressive effects on MDD patients. Anti-inflammatory agents have shown positive therapeutic potential for treating MDD patients, as confirmed by several meta-analyses. In addition, anti-inflammatory agents and their combination with antidepressants are more effective in treating MDD, with fewer side effects. Bai and co-workers demonstrated that adjunctive treatment exhibited an increased effect size compared with monotherapy, and there was a significant difference in MDD severity between baseline and endpoint (Bai et al., 2020). This indicates that adjunctive treatment is more effective than monotherapy. Furthermore, it has been suggested that anti-inflammatory agents play an antidepressive role in MDD, and are considered to be safe (Köhler-Forsberg et al., 2019).

Previous studies have analyzed the efficacy of anti-inflammatory agents in MDD patients. However, the comparison among different anti-inflammatory agents for the treatment of MDD is still lacking. In routine practice, clinical practitioners have a broad range of therapeutic choices and they require strong evidence to decide the best treatment for each individual patient (Cipriani et al., 2018). Therefore, we conducted a network meta-analysis (NMA) to compare different anti-inflammatory agents for improving the treatment of MDD patients.

## Materials and Methods

The NMA protocol was registered with PROSPERO (CRD42020182531), and was performed in compliance with the Preferred Reporting Items for Systematic Reviews and Meta-Analyses (PRISMA) extension statement (Moher et al., 2009; Hutton et al., 2015).

### Search Strategy

To identify eligible randomized clinical trials, four databases including the Cochrane Library, Web of Science, PubMed and Embase were searched from inception date to May 31, 2020. The trial registers in ClinicalTrials.gov and World Health Organization were also searched. The articles published in English were selected. A combination of free words and subject words was used when searching the electronic databases. Considering that the term "anti-inflammatory agents" is a broad concept, we paid much attention to some important reviews regarding the effects of anti-inflammatory agents on MDD treatment (Köhler et al., 2016; Köhler-Forsberg et al., 2019; Bai et al., 2020). The articles reporting on modafinil were removed, as it exhibited certain abuse/addictive potential and was tightly restricted in some countries (Kumar, 2008; Davies et al., 2013). Non-steroidal anti-inflammatory drugs (NSAIDs), corticosteroids, cytokine inhibitors, statins, pioglitazone, minocycline, N-acetylcysteine (NAC) and omega-3 fatty acid (Omega-3 FA) were all regarded as anti-inflammatory agents. The following search terms were used: nonsteroidal anti-inflammatory drug (combined with NSAID, COX-2 inhibitor, COX inhibitor, cyclooxygenase 2 inhibitor, cyclooxygenase inhibitor, aspirin, acetylsalicylic acid, acetaminophen, paracetamol, diclofenac, ibuprofen, rofecoxib and celecoxib); corticosteroid (combined with glucocorticoid, prednisone, meprednisone, prednisolone, hydrocortisone and dexamethasone); cytokine inhibitor (combined with TNF inhibitor, tumour necrosis factor inhibitor, infliximab, etanercept, adalimumab and ustekinumab); statin (combined with HMG-CoA reductase inhibitor, lipid-lowering agent, lipid-lowering drug, pravastatin, simvastatin, atorvastatin, rosuvastatin, fluvastatin, lovastatin and pitavastatin); minocycline, pioglitazone, omega-3 fatty acid (combined with docosahexaenoic acid, eicosapentaenoic acid, poly-unsaturated fatty acid, DHA, EPA and PUFA), N-acetylcysteine (combined with NAC), major depressive disorder (combined with depression and major depression), randomized clinical trial (combined with randomized control trial and random). The reference lists of relevant meta-analysis, pooled analysis, reviews and included studies were also checked to find additional studies. Unpublished clinical trials were excluded due to the unreliability of the data. More details on the search strategies can be found in Supplementary Table S1.

### Selection Criteria

The studies were screened for the following inclusion criteria: (i) A randomized, placebo-controlled, clinical trial assessing the efficacy and acceptability of anti-inflammatory agent as monotherapy (anti-inflammatory agent vs. placebo) or as combination therapy (anti-inflammatory agent + antidepressant agent vs. placebo + antidepressant agent) in patients with MDD; (ii) these patients (aged ≥18 years) were diagnosed based on any recognized diagnostic criteria such as Diagnostic and Statistical Manual of Mental Disorders, Fifth Edition (DSM-V) or International Classification of Diseases, 10th Revision (ICD-10); (iii) the severity of depressive symptoms was evaluated by the Montgomery-Asberg Depression Rating Scale (MADRS), Hamilton Rating Scale for Depression (HAMD), Geriatric Depression Scale (GDS) or Beck’s Depression Inventory-II (BDI-II); and (iv) only the trial with the largest number of patients or most comprehensive information was included if overlapping data were published by the same research group. The studies were also screened for the following exclusion criteria: (i) anti-inflammatory agent trials in depressive patients with a severe concomitant disease or females with postpartum depression; (ii) the studies only reported on bipolar depression, juvenile depression or seasonal depression as well as those focused on adverse events or costs; (iii) the studies published as scientific meeting abstracts or conference proceedings; and (iv) the trials with no outcome indicators.

### Outcome Assessment

Efficacy, acceptability and remission rate were the main outcomes in this NMA. The efficacy of anti-inflammatory agents was assessed by the treatment response rate, which defined as the number of patients who exhibit ≥50% reduction of depressive symptoms (Furukawa et al., 2016). The acceptability of anti-inflammatory agents was measured by all-cause treatment discontinuation, as it encompassed both efficacy and tolerability (Cipriani et al., 2009). The definition of remission rate was as follows: MADRS ≤7, HAMD ≤7, GDS ≤11 or BDI-II ≤8 at the end stage of the trial (Bai et al., 2020).

### Date Extraction

All identified studies were imported into Endnote X9. First, we removed duplicate studies. Subsequently, two independent reviewers screened the title and abstract of each article. After that, the full texts of related studies were reviewed based on the selection criteria. In case of any disagreement, the final decision was made by the third reviewer. The study information (author name, date of publication and sample size), patient characteristics (age, gender, diagnostic criteria and disease course), intervention details (intervention type, treatment duration and effectiveness of interventions and placebo) and clinical outcomes were recorded.

### Risk of Bias Assessment

Two independent reviewers used the Review Manager 5.3 to evaluate the quality of the included randomized clinical trials according to the Cochrane risk-of-bias assessment tool (Higgins et al., 2011). The following aspects were examined: (i) allocation concealment, (ii) sequence generation, (iii) blinding of outcome assessment, (iv) blinding of participants and personnel, (v) selective reporting, (vi) incomplete outcome data, and (vii) other bias.

### Data Analysis

Review Manager 5.3 was used to conduct the conventional pairwise meta-analysis for determining the effects of different anti-inflammatory agents. In this NMA, we employed the STATA 14.0 ("mvmeta" and "network" packages) to draw the trial network plots and assess for publication bias and R 3.6.3 ("ggplot2", "JAGS" and "gemtc" packages) to conduct statistical analysis. The R 3.6.3 was employed for a Bayesian frame structure, while STATA 14.0 was used in a frequentist framework. Risk ratio (RR) with confidence interval (CI) of 95% was adopted as a representative measure of dichotomous outcomes. The level of statistical significance was set as *p* < 0.05.

For the conventional pairwise meta-analysis, heterogeneity among studies was estimated by I-squared (*I* ^2^) tests and Cochran’s Q test. Based on the Cochrane Collaboration Handbook, the *I* ^2^ values of 75, 50, and 25% indicate high, moderate and low heterogeneity, respectively. When a moderate or high heterogeneity (*I* ^2^ > 50% and *p*-value < 0.1) was observed, a random-effect model was employed; otherwise, a fixed-effect model was applied (Higgins et al., 2003).

For the NMA, the analysis was conducted in a Bayesian framework. Markov chain Monte Carlo method was used to compute an effect measure for each anti-inflammatory agent. A convergence diagnostic plot was constructed using the Brooks-Gelman-Rubin statistics, with 50,000 adaptation iterations for obtaining convergence and 100,000 simulation iterations (thinning factor = 10) for generating the outputs (Gelman and Rubin, 1992; BROOKS.and GELMAN, 1998). The analysis was conducted under a random effect model to explain the between-study heterogeneity such as clinical heterogeneity and produce more generalizable results (Shi et al., 2020). Residual deviance represents the contribution of 1 data point for each study arm in a well-fitting model. The smaller the deviance, the better the fit (Spiegelhalter et al., 2002). Therefore, the value of residual deviance was employed to assess the model fit between random model and fixed model. Node-splitting analysis was performed to evaluate the consistency between indirect and direct comparisons, and a *p*-value of <0.05 was regarded as inconsistent (van et al., 2016). The potential scale reduced factor (PSRF) value of ∼1 implies that the results have good convergence and the consistency model is considered to be roust. We also used the trace plot and density plot to assess convergence. Surface under the cumulative ranking curve (SUCRA) is a representative number of the overall ranking, and a lower SUCRA value denotes a lower probability (Daly et al., 2019; Hoang and Kim, 2020). We ranked interventions by calculating the values of SUCRA. We also calculated a ratio to obtain the decreased amount of RR (acceptability) per one unit of RR (efficacy) for each anti-inflammatory agent by comparing with the placebo group (Hoang et al., 2020). Finally, a meta-regression analysis was carried out.

## Results

### Characteristics of the Included Studies

**FIGURE 1:**
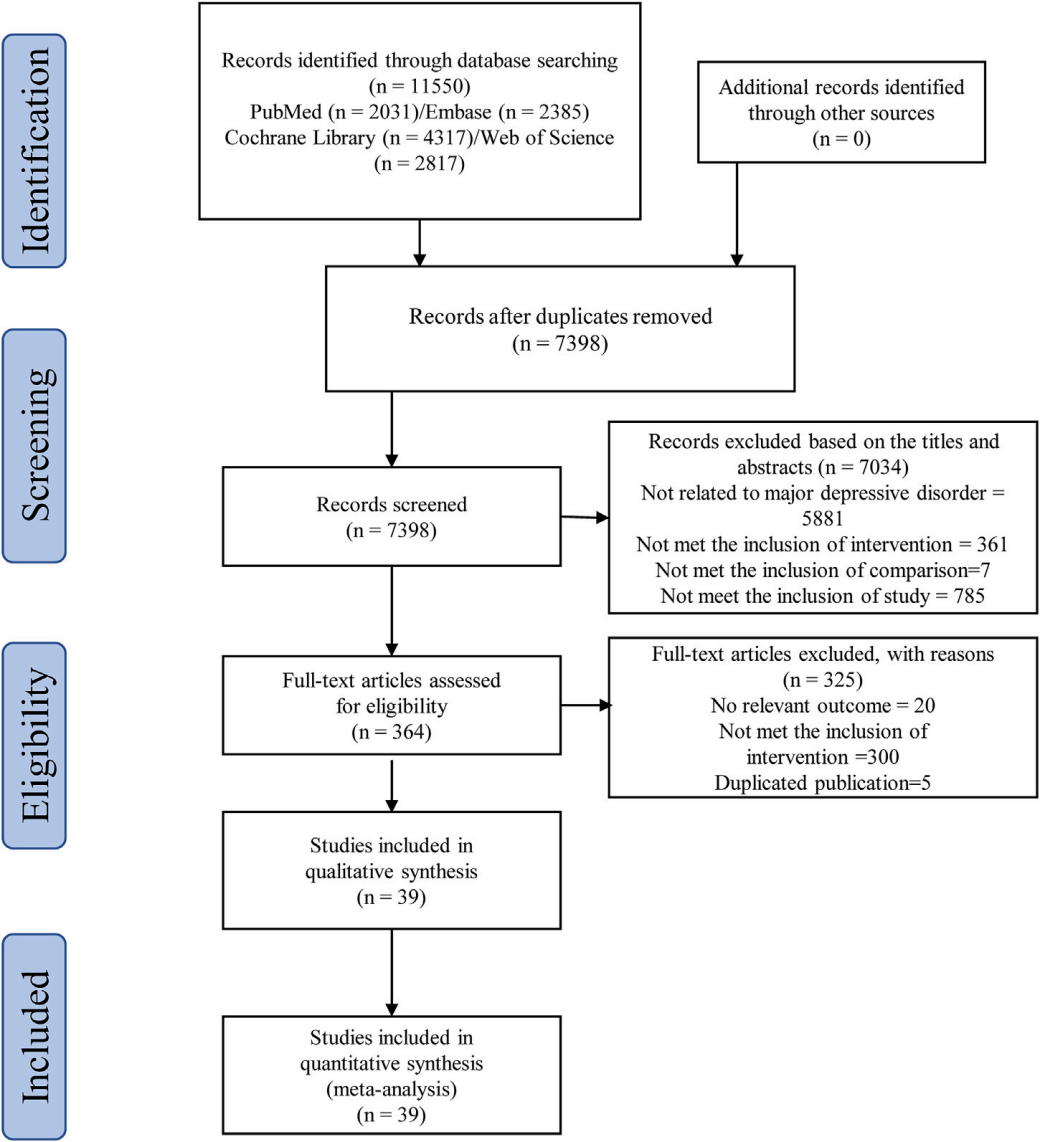
Literature search and selection.

According to the search strategy of this study, a total of 11,550 related studies were obtained in the initial examination. After screening titles/abstracts and removing duplicate studies, the full texts of 364 potentially eligible studies were obtained. Ultimately, 39 randomized clinical trials (Akhondzadeh et al., 2009; Abbasi et al., 2012; da Silva et al., 2008; Krause et al., 2017; Majd et al., 2015; Müller et al., 2006; Sepehrmanesh et al., 2017; Arana et al., 1995; DeBattista et al., 2000; Otte et al., 2010; Ghanizadeh and Hedayati, 2013; Gougol et al., 2015; Haghighi et al., 2014; Rasgon et al., 2016; Sepanjnia et al., 2012; Dean et al., 2017; Emadi-Kouchak et al., 2016; Husain et al., 2017; Berk et al., 2014; Bot et al., 2010; Carney et al., 2009; Carney et al., 2019; Chang et al., 2020; Gertsik, 2012; Grenyer et al., 2007; Jahangard et al., 2018; Jazayeri et al., 2008; Jiang et al., 2018; Keshavarz et al., 2018; Lespérance et al., 2011; Marangell et al., 2003; Mischoulon et al., 2009; Mischoulon et al., 2015; Nemets et al., 2002; Park et al., 2015; Rapaport et al., 2016; Rondanelli et al., 2010; Shinto et al., 2016; Su et al., 2003) were included in the quantitative data synthesis. Figure 1 illustrates the systematic literature searching and study selection processes. The characteristics of the included trials are summarized in Table 1. These studies were all published in English journals between 1995 and 2019 years. There were 2,871 participants reported in these studies. All of these studies were of placebo controlled. Different interventions were applied in the 39 randomized clinical trials, including NSAIDs (*n* = 6), corticosteroids (*n* = 3), statins (*n* = 3), pioglitazone (*n* = 2), minocycline (*n* = 3), NAC (*n* = 1) and Omega-3 FA (*n* = 21). The sample sizes of the included trials ranged from 20 to 432. The medication doses were flexible in 7 trials. The mean ages of adult MDD patients ranged from 20 to 84.9 years. The study duration ranged between 2 days and 16 weeks. Twenty-three out of 39 trials (58.97%) were funded by non-profit organization.

**TABLE 1:** Characteristics of included studies.

Included studies	Diagnostic criteria	Sample size	Experimental group			Control group			Follow-up time (weeks)	Sponsor type
			Mean age (Years)	Female	Interventions	Mean age (Years)	Female	Interventions		
NSAIDs										
Abbasi et al. (2012)	DSM-IV-TR	40	35.1 ± 8.0	7	Sertraline 200 mg/d + celecoxib 400 mg/d	34.2 ± 6.9	6	Sertraline 200 mg/d + placebo	6	NPO
Akhondzadeh et al. (2009)	DSM-IV	40	34.65 ± 6.76	13	Fluoxetine	34.20 ± 4.96	12	Fluoxetine	6	NPO
					20–40 mg/d + celecoxib			20–40 mg/d + placebo		
					400 mg/d					
Krause et al. (2017)	DSM-IV	32	44.6 ± 11.5	7	Reboxetine 4–10 mg/d + celecoxib 400 mg/d	43.9 ± 13.3	9	Reboxetine 4–10 mg/d + placebo	6	NPO
Majd et al. (2015)	DSM-IV-TR	30	34.7 ± 7.3	15	Sertraline 200 mg/d + celecoxib 200 mg/d	36.2 ± 12.7	15	Sertraline 200 mg/d + placebo	8	NC
Müller et al. (2006)	DSM-IV	40	44.5 ± 11.6	8	Reboxetine 4–10 mg/d + celecoxib 200–400 mg/d	44.3 ± 13.5	12	Reboxetine 4–10 mg/d + placebo	6	CI
Sepehrmanesh et al. (2017)	DSM-IV	100	48.9 ± 7.5	29	Sertraline 50–200 mg/d + aspirin 16.mg/d	47.8 ± 7.3	32	Sertraline 50–200 mg/d + placebo	8	NPO
Corticosteroids										
Arana et al. (1995)	DSM-III-R	37	20–67	NA	Dexamethasone 4 mg/d	20–67	NA	Placebo	4 days	NPO
Debattista et al. (2000)	DSM-III-R	22	46.7 ± 18/35 ± 10.5	6	Ovine CRH 1 ug/kg/ Hydrocortisone 15 mg	39.8 ± 10.1	7	Placebo	2 days	NPO
Otte et al. (2010)	DSM-IV	37	36.5 ± 12.7	15	Escitalopram 10 mg/d + Fludrocortisone 0.2 mg/d	34.5 ± 12.7	8	Escitalopram 10 mg/d + placebo	3	CI
Statins										
Ghanizadeh and Hedayati, (2013)	DSM-IV	60	32.5 ± 10.2	22	Fluoxetine 40 mg/d + lovastatin 30 mg/d	31.7 + 9.3	21	Fluoxetine 40 mg/d + placebo	6	NC
Gougol et al. (2015)	DSM-IV-TR	44	36.4 ± 8.1	13	Fluoxetine 20 mg/d + Simvastatin 20 mg/d	34.2 ± 10.8	16	Fluoxetine 20 mg/d + placebo	6	NPO
Haghighi et al. (2014)	DSM-V	60	33.07 ± 8.85	14	Citalopram 40 mg/d + atorvastatin 20 mg/d	31.43 ± 7.96	14	Citalopram 40 mg/d + placebo	12	NPO
Pioglitazone										
Rasgon et al. (2016)	DSM-IV	42	49.42	17	Pioglitazone 30 mg + TAU	43.28	16	Placebo + TAU	12	NPO
Sepanjnia et al. (2012)	DSM-IV-TR	40	31.4 ± 5.4	14	Citalopram 20–30 mg/d + pioglitazone 30 mg	32.7 ± 5.4	15	Citalopram 20–30 mg/d + placebo	6	NPO
Minocycline										
Dean et al. (2017)	DSM-IV	71	51.0 ± 14.6	24	Minocycline 200 mg/d + TAU	47.8 ± 14.8	23	Placebo + TAU	12	NPO
Emadi-Kouchak et al. (2016)	DSM-IV-TR	46	34.70 ± 7.43	9	Minocycline 200 mg/d	36.35 ± 8.00	7	Placebo	6	NPO
Husain et al. (2017)	DSM-V	41	40 (30–46)	11	Minocycline 200 mg/d + TAU	35 (30.5–39)	10	Placebo + TAU	12	NPO
NACs										
Berk et al. (2014)	DSM-IV-TR	252	49.9 ± 13	84	N-acetylcysteine 2 g/d + TAU	50.5 ± 12.5	75	Placebo + TAU	12	NPO
Omega-3 FA										
Bot et al. (2010)	DSM-IV	25	53.1 ± 13.8	8	E-EPA 1 g/d + TAU	55.0 ± 8.6	5	Placebo + TAU	12	CI
Carney et al. (2009)	DSM-IV	122	58.1 ± 9.4	22	Sertraline 50 mg/d + omega-3 2 g/d	58.6 ± 8.5	19	Sertraline 50 mg/d + placebo	10	CI
Carney et al. (2019)	DSM-V	144	58.5 ± 9.6	26	Sertraline 50 mg/d + EPA 2 g/d	60.5 ± 9.3	30	Sertraline 50 mg/d + placebo	10	NPO
Chang et al. (2020)	DSM-IV/ICD-10	59	61.10 ± 9.14	12	EPA 2 g + DHA 1 g	61.93 ± 8.95	9	Placebo	12	NPO
Gertsik (2012)	DSM-IV	40	NA	NA	Citalopram 20–40 mg/d + n-3 PUFA 1.2 g	NA	NA	Citalopram 20–40 mg/d + placebo	9	CI
Grenyer et al. (2007)	DSM-IV	83	NA	NA	n-3 PUFA 3 g + TAU	NA	NA	Placebo + TAU	16	CI
Jahangard et al. (2018)	DSM-V	50	41.28 ± 11.56	8	Sertraline 50–200 mg/d + n-3 PUFA 1 g	43.64 ± 11.29	8	Sertraline 50–200 mg/d + placebo	12	NPO
Jazayeri et al. (2008)	DSM-IV	60	34.5 ± 11.3	9	Fluoxetine 20 mg + 1 g EPA	35.1 ± 9.4	12	Fluoxetine 20 mg + placebo	8	NPO
Jiang et al. (2018)	DSM- IV	72	57.73 ± 16.14	15	n-3 PUFA 2 g	57.91 ± 11.68	23	Placebo	12	NC
Keshavarz et al. (2018)	DSM-V	65	41 ± 9.9	NA	EPA 1.08 g + DHA 0.72 g	44 ± 9.5	NA	Placebo	12	NPO
Lespérance et al. (2011)	MINI	432	46.6 ± 11.54	143	EPA 1.05 g + DHA 0.15 g	45.4 ± 13.27	153	Placebo	8	CI
Marangell et al. (2003)	DSM-IV	36	46.8 ± 11.6	14	DHA 2 g/d	47.9 ± 11.2	14	Placebo	6	CI
Mischoulon et al. (2009)	DSM-IV	57	43 ± 13	16	EPA 1 g/d	43 ± 13	19	Placebo	8	NC
Mischoulon et al. (2015)	DSM-IV	196	46.2 ± 11.8/46.3 ± 13.7	70	EPA 1 g/d/ DHA 1 g/d	45.0 ± 12.1	35	Placebo	8	NPO
Nemets et al. (2002)	DSM-IV	20	54.2 ± 13.9	9	E-EPA 2 g/d + TAU	52.1 ± 10.2	8	Placebo + TAU	4	NC
Park et al. (2015)	DSM-IV	35	43.50 ± 3.72	14	EPA 1.14 g + DHA 0.6 g + TAU	39.41 ± 3.58	13	Placebo + TAU	12	NPO
Rapaport et al. (2016)	DSM-IV	155	46.1 ± 12.6	NA	EPA 1.06 g + DHA 0.26 g/ EPA 0.18 g/ DHA 0.9 g	46.1 ± 12.6	NA	Placebo	8	NC
Rondanelli et al. (2010)	DSM-IV-TR	46	84.9 ± 6.9	22	n-3 PUFA 2.5 g/d	83.0 ± 7.3	24	Placebo	8	NPO
Shinto et al. (2016)	DSM-IV	31	50.7 ± 11.6	19	EPA 1.95 g/d + DHA 1.35 g/d	51.9 ± 10	17	Placebo	12	NC
Da Silva et al. (2008)	DSM-IV	31	64.4	NA	EPA 0.72 g/d + DHA 0.48 g/d/ EPA 0.72 g/d + DHA 0.48 g/d + TAU	64.4	NA	Placebo/placebo + TAU	12	NPO
Su et al. (2003)	DSM-IV	22	35.2 ± 11.6	10	Omega-3 PUFAs 9.6 g/d + TAU	42.3 ± 10.7	8	Placebo + TAU	8	CI

CI: commercial industry; DHA: Docosahexaenoic Acid; DSM: Diagnostic and Statistical Manual for Mental Disorders; EPA: Eicosapentaenoic Acid; ICD: International Classification of Diseases; MINI: Mini-International Neuropsychiatric Interview; NC: not clear; NPO: non-profit organization; PUFA: polyunsaturated fatty acid; TAU: treatment as usual.

### Risk of Bias

Twenty-five (64.1%) randomized clinical trials exhibited a low risk of bias for inadequate sequence generation. With regard to allocation concealment, 33 (84.6%) trials had a low risk, which adopted opaque envelope or the central randomization system. In terms of blind methods, 2 (5.1%) trials had no blinding of participants and personnel, while 4 (10.2%) trials had no blinding of outcome assessments. All randomized clinical trials had a low risk of selective reporting bias and incomplete outcome data. Other bias was unclear in all the included randomized clinical trials. Supplementary Figures S1 and S2 illustrate the summary assessments of the risk of bias.

### Pairwise Meta-Analysis

Twenty-one studies reported the response rates that could reflect the effects of anti-inflammatory agents on MDD patients. As shown in Supplementary Figure S3A, pooling analysis revealed that anti-inflammatory agents exerted considerable antidepressant-like effects (RR = 1.41, 95%CI: 1.17–1.68, *p* = 0.0002). Heterogeneity among studies was found to be moderate (X^2^ = 44.54, df = 21, *p* = 0.002, I^2^ = 53%).

Thirty-eight studies demonstrated the acceptability of each anti-inflammatory agent on MDD patients. As shown in Supplementary Figure S3B, the differences in acceptance rates between anti-inflammatory agents and placebo were not statistically significant (RR = 1.02, 95%CI: 0.99–1.05, *p* = 0.26). No heterogeneity was observed among studies (X^2^ = 29.79, df = 38, *p* = 0.83, I^2^ = 0%).

Remission rates were reported in 16 studies involving 5 inflammatory agents. As shown in Supplementary Figure S3C, the remission rates were markedly reduced after treatment with anti-inflammatory agents (RR = 1.54, 95%CI: 1.14–2.07, *p* = 0.004). The degree of heterogeneity among studies was relatively low (X^2^ = 23.45, df = 14, *p* = 0.05, I^2^ = 40%).

### Network Meta-Analysis

**FIGURE 2:**
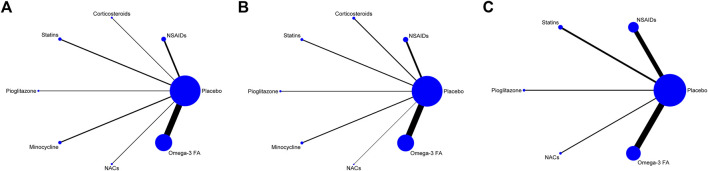
**(A)** Network map for efficacy. **(B)** Network map for acceptability. **(C)** Network map for remission. Display of the network of eligible studies for efficacy **(A)** and acceptability **(B)** and remission **(C)**. The width of the line indicates the number of trials comparing two agents. The size of the node indicates the number of MDD patients randomized to a particular agent. NSAIDs: non-steroidal anti-inflammatory drugs; NACs: N-acetylcysteines; Omega-3 FA: omega-3 fatty acid.

Trial network plots are shown in Figure 2A–C. The width of the line indicates the number of trials comparing two agents. The size of the node indicates the number of MDD patients randomized to a particular agent. It was found that the samples of placebo and Omega-3 FA ranked the highest in this NMA. However, there was no direct comparison between any two anti-inflammatory agents, and they were all compared with placebo group. Hence, this NMA was carried out to evaluate both direct and indirect comparisons.

Since no closed loop was formed in each network graph, the possible inconsistencies in NMA were not tested and only the consistency model was selected. According to the values of residual deviance (Supplementary Table S2), random model was found to be relatively better than fixed model. The Brooks–Gelman–Rubin diagnostic plot revealed the median value of the scale reduction parameter and 97.5% tended to be stable following 50,000 iterations. Subsequently, Bayesian models were employed for a 100,000 iterative calculation. The value of PSRF was close to 1, indicating a satisfactory convergence (Supplementary Figure S4A–C). Furthermore, we constructed density plot and trace plot (Supplementary Figure S5A1–C2), and the results also indicated a satisfactory convergence.

Tables 2, 3 and 4 summarizes the NMA comparison results of efficacy, acceptability and remission. For efficacy, NSAIDs (RR = 0.50, 95%CI: 0.26–0.73) and pioglitazone (RR = 0.45, 95%CI: 0.20–0.84) were more favorable than placebo. Meanwhile, NSAIDs had a significant higher efficacy than Omega-3 FA (RR = 1.87, 95%CI: 1.18–3.52), and pioglitazone was superior to Omega-3 FA (RR = 2.08, 95%CI: 1.03–4.55). With respect to acceptability, NSAIDs were more acceptable than placebo (RR = 0.89, 95%CI: 0.77–0.99) and minocycline (RR = 1.22, 95%CI: 1.03–1.49). For remission, NSAIDs were more superior than placebo (RR = 0.48, 95%CI: 0.27–0.79) and Omega-3 FA (RR = 2.01, 95%CI: 1.09–3.90). NACs were more favorable than placebo (RR = 0.39, 95%CI: 0.13–0.99).

**TABLE 2:** Network meta-analysis comparisons for efficacy.

Placebo							
**0.50 (0.26, 0.73)**	NSAIDs						
0.25 (0.03, 1.02)	0.52 (0.07, 2.37)	Corticosteroids					
0.68 (0.38, 1.18)	1.37 (0.70, 3.24)	2.67 (0.59, 22.43)	Statins				
**0.45 (0.20, 0.84)**	0.91 (0.39, 2.22)	1.75 (0.36, 14.73)	0.66 (0.25, 1.54)	Pioglitazone			
0.45 (0.17, 1.08)	0.93 (0.32, 2.73)	1.79 (0.32, 16.16)	0.67 (0.22, 1.92)	1.02 (0.32, 3.23)	Minocycline		
0.65 (0.35, 1.17)	1.31 (0.65, 3.19)	2.57 (0.56, 21.56)	0.96 (0.42, 2.17)	1.45 (0.60, 3.84)	1.43 (0.49, 4.51)	NACs	
0.93 (0.71, 1.11)	**1.87 (1.18, 3.52)**	3.64 (0.88, 28.67)	1.38 (0.72, 2.45)	**2.08 (1.03, 4.55)**	2.05 (0.81, 5.48)	1.44 (0.73, 2.63)	Omega-3 FA

Data are RRs (95% CI) in the column-defining treatment compared with the row-defining treatment. RRs higher than 1 favour the column-defining treatment. RRs lower than 1 favour the row-defining treatment. Significant results are in bold. NSAIDs: non-steroidal anti-inflammatory drugs; NACs: N-acetylcysteines; Omega-3 FA: omega-3 fatty acid.

**TABLE 3:** Network meta-analysis comparisons for acceptability.

Placebo							
**0.89 (0.77, 0.99)**	NSAIDs						
0.89 (0.64, 1.04)	1.00 (0.72, 1.23)	Corticosteroids					
0.97 (0.87, 1.08)	1.09 (0.93, 1.3)	1.09 (0.9, 1.54)	Statins				
0.94 (0.75, 1.11)	1.06 (0.83, 1.31)	1.06 (0.8, 1.52)	0.97 (0.76, 1.18)	Pioglitazone			
1.08 (0.95, 1.25)	**1.22 (1.03, 1.49)**	1.22 (0.99, 1.72)	1.11 (0.94, 1.34)	1.15 (0.93, 1.51)	Minocycline		
0.92 (0.79, 1.08)	1.04 (0.86, 1.28)	1.04 (0.83, 1.48)	0.95 (0.78, 1.15)	0.98 (0.78, 1.29)	0.85 (0.69, 1.05)	NACs	
0.98 (0.93, 1.02)	1.10 (0.99, 1.28)	1.10 (0.94, 1.52)	1.01 (0.90, 1.14)	1.04 (0.88, 1.31)	0.91 (0.78, 1.04)	1.07 (0.90, 1.25)	Omega-3 FA

Data are RRs (95% CI) in the column-defining treatment compared with the row-defining treatment. RRs higher than 1 favour the column-defining treatment. RRs lower than 1 favour the row-defining treatment. Significant results are in bold. NSAIDs: non-steroidal anti-inflammatory drugs; NACs: N-acetylcysteines; Omega-3 FA: omega-3 fatty acid.

**TABLE 4:** Network meta-analysis comparisons for remission.

Placebo					
**0.48 (0.27, 0.79)**	NSAIDs				
0.87 (0.42, 1.91)	1.81 (0.75, 4.93)	Statins			
0.40 (0.11, 1.20)	0.83 (0.21, 2.92)	0.46 (0.10, 1.70)	Pioglitazone		
**0.39 (0.13, 0.99)**	0.81 (0.25, 2.45)	0.44 (0.12, 1.47)	0.97 (0.21, 4.77)	NACs	
0.97 (0.67, 1.31)	**2.01 (1.09, 3.90)**	1.12 (0.46, 2.43)	2.42 (0.75, 9.03)	2.51 (0.90, 7.45)	Omega-3 FA

Data are RRs (95% CI) in the column-defining treatment compared with the row-defining treatment. RRs higher than 1 favour the column-defining treatment. RRs lower than 1 favour the row-defining treatment. Significant results are in bold. NSAIDs: non-steroidal anti-inflammatory drugs; NACs: N-acetylcysteines; Omega-3 FA: omega-3 fatty acid.

Supplementary Figure S6 visually shows two-dimensional graphs reporting the RR values of efficacy and acceptability when compared to placebo group. The results indicated that the RR reduction of acceptability was obtained per 1 unit RR of efficacy for NSAIDs (0.55), corticosteroids (0.28), statins (0.69), pioglitazone (0.48), minocycline (0.42), NAC (0.70) and Omega-3 FA (0.95). Based on the results of SUCRA value (Table 5), corticosteroids were the best anti-inflammatory agent for MDD patients due to their high efficacy. As for acceptability, NSAIDs were much better than other anti-inflammatory agents. Besides, NACs were the best anti-inflammatory agent in the terms of remission. The comparison-adjusted funnel plots are displayed in Supplementary Figure S7A–C, and the results demonstrated no robust evidence of small-study effects for each outcome among the included studies.

**TABLE 5:** SUCRA value for treatment ranking.

Treatment	Efficacy	Acceptability	Remission
Placebo	0.06	0.26	0.17
NSAIDs	0.67	**0.81**	0.72
Corticosteroids	**0.86**	0.77	—
Statins	0.41	0.45	0.31
Pioglitazone	0.71	0.58	0.77
Minocycline	0.68	0.07	—
NAC	0.45	0.65	**0.80**
Omega-3 FA	0.16	0.41	0.22

The surface under the cumulative ranking curve (SUCRA) value is a representative number of the overall ranking and a higher SUCRA value indicates a higher probability. NSAIDs: non-steroidal anti-inflammatory drugs; NAC: N-acetylcysteine; Omega-3 FA: omega-3 fatty acid. The highest values of SUCRA are in bold.

### Meta-Regression Analysis

Three covariates, such as monotherapy or adjunctive therapy, duration of treatment and gender (only include women or include men and women), were selected to conduct a meta-regression analysis of the outcomes. There were no covariates showing a significant coefficient in the interaction model. All results are presented in Supplementary Table S3.

### Safety of Anti-Inflammatory Agents

A previous study reported 5 serious adverse events in the NAC group and 4 serious adverse events in the placebo group, but no significant difference was observed between the two groups (Berk et al., 2014). NAC group had a remarkably higher percentage of gastrointestinal problems compared to placebo group (Berk et al., 2014). Another study reported 7 serious adverse events in the Omega-3 FA group and 4 serious adverse events in the placebo group (Lespérance et al., 2011). However, no serious adverse events were reported in other studies. We conducted quantitative data synthesis on 13 kinds of non-serious adverse events (abdominal pain, anxiety, constipation, decreased appetite, diarrhea, dyspepsia, headache, increased appetite, insomnia, nausea, sexual dysfunction, sweating and tremor) among 16 studies. As shown in Supplementary Figure S8, the patients in Omega-3 FA group had a lower incidence of anxiety and decreased appetite compared to those in control group. Besides, the patients in control group exhibited a higher incidence of insomnia and sweating compared to those in minocycline group. Apart from the above, there was no difference in the rates of adverse events between placebo and anti-inflammatory agent groups.

## Discussion

Inflammatory cytokines play crucial roles in the development and progression of MDD through neurotransmission, neuroplasticity and neuroendocrine processes. Anti-inflammatory agents can exert antidepressive effects on MDD by mediating neuroplasticity genes, neurotransmitter systems and glucocorticoid receptor pathway (Adzic et al., 2018). Several meta-analyses have been published, but the comparison among different anti-inflammatory agents for MDD treatment is still lacking. To our knowledge, this NMA constituted the best available evidence about the comparisons of efficacy for each anti-inflammatory agent. Therefore, the results of this NMA might help doctors in making clinical decisions.

In the present study, priority was given to the assessment of dichotomous outcomes for efficacy, acceptability and remission rate. This is because the clinical trials of antidepressant agents have a small sample size and it is difficult to evaluate the data distribution of these small studies (Furukawa et al., 2016). In the pairwise meta-analysis, we found that the anti-inflammatory agent group had higher response rate (efficacy) and remission rate than the placebo group. For acceptability, no obvious difference was found between the two groups, indicating that MDD patients can benefit from the anti-inflammatory agents without increasing the risk of side effects. These results are consistent with the findings of two previous meta-analyses (Köhler-Forsberg et al., 2019; Bai et al., 2020). In the NMA comparisons for the anti-inflammatory interventions, we found that corticosteroids might have advantages over other agents in terms of efficacy (which was measured by response rate according to the SUCRA value). However, the head-to-head comparisons for the efficacy of glucocorticoids and other agents were not statistically significant. Therefore, this result must be interpreted with caution. Glucocorticoid receptor may be a therapeutic target for MDD patients because it is involved in both immune regulation and depression. Corticosteroids possibly restore the negative feedback loop on the hypothalamic–pituitary–adrenal axis to exhibit their antidepressive effects (Sun et al., 2016). In a previous trial, the response rate of dexamethasone group could achieve 37%, while that of placebo group was only 6% (Arana et al., 1995). With regard to acceptability (which was measured by all-cause treatment discontinuation), NSAIDs became our recommendation. This anti-inflammatory agent can act on MDD by suppressing COX-1 and COX-2 that are required for the production of inflammation-associated prostaglandins. It is worth mentioning that COX-2 inhibitor has a direct effect on serotonergic neurons in the CNS (Müller, 2019). With respect to remission, NAC was considered as the best antidepressive agent among the five studied anti-inflammatory agents. NAC is a multi-target molecule and it can decrease neuroinflammation by inhibiting microglia that contributes to the occurrence and progression of inflammatory responses in MDD patients (Roman and Irwin, 2020).

Apart from the above three agents, other agents also demonstrated excellent antidepressive effects. In the CNS, statins can induce the activation of microglia and astrocytes, as well as the release of cytokines by inhibiting NF-kB signaling and subsequent TNF-α, IL-1β and IL-6 production (Lim et al., 2017; Taniguti et al., 2019; Yu et al., 2019). Besides, the microglia-mediated inflammatory response can be suppressed by regulating microglial polarization under the action of pioglitazone (Essmat et al., 2020). It has also been reported that minocycline can inhibit neurotoxic factors released by microglia and induce neuroprotective activities released by astrocytes in order to exert its antidepressive effects (Soczynska et al., 2012). Furthermore, the antidepressive effect of Omega-3 FA can be explained by several key mechanisms such as neurotransmitter dysregulation, neuroplasticity and neuroinflammatory processes (Trebaticka and Durackova, 2014).

In the meta-regression analysis, the effects of monotherapy, adjunctive therapy, duration and gender were examined. It was found that these factors did not influence the NMA results. Although there was no statistical significance about efficacy, we still recommended adjunctive therapy because the effect value of adjunctive therapy was larger than that of monotherapy. This opinion is supported by a previous meta-analysis showing that adjunctive therapy has a larger effect size than monotherapy (Bai et al., 2020). Furthermore, antidepressants are probably irreplaceable in the treatment of MDD (Fournier et al., 2010).

Nowadays, anti-inflammatory agents may become a new treatment opinion for MDD patients because of the positive association between depression and inflammatory processes (Köhler et al., 2016). In this study, we found that almost all the included trials had focused on the antidepressive effect of one specific type or subtype of anti-inflammatory agent. Only few studies explored the effect of the combination of different anti-inflammatory agents. A trial involving 24 patients reported that the co-administration of NAC and aspirin could alleviate depressive symptoms after 16 weeks, which was remarkably better than NAC or aspirin treatment alone (Bauer et al., 2018). In another trial, no evidence was found that minocycline plus celecoxib was more effective than placebo for treating depression (Husain et al., 2020). However, these two studies were concerned on bipolar depression, and there was no direct evidence for the combined effect of different anti-inflammatory agents on MDD treatment. This might be a meaningful research direction, which can serve as a reference for further clinical trials. Besides, we found that the same anti-inflammatory agent might enhance the efficacy regardless of types of antidepressants. For example, celecoxib combined with sertraline or fluoxetine could exhibit better antidepressive effect than sertraline or fluoxetine treatment alone (Akhondzadeh et al., 2009; Abbasi et al., 2012). Taking account into different types of antidepressants and small number of the included studies, whether the same anti-inflammatory agent can enhance the efficacy of multiple antidepressants deserves to be further explored. In this NMA, we evaluated the incidence rates of 13 types of non-serious adverse events, and found that all anti-inflammatory agents demonstrated a good safety profile.

Nevertheless, our study has some limitations. First, for the selection of anti-inflammatory agents, we did not cover all anti-inflammatory agents but instead the main anti-inflammatory agents that could be used for MDD treatment. Minocycline is a type of antibiotics and pioglitazone is a type of thiazolidinedione. Although we performed an internet search using the terms "antibiotics" and "thiazolidinedione" and found the current trials of minocycline or pioglitazone in MDD patients, it was possible to neglect some useful information regarding "minocycline" and "pioglitazone" in the final formal search. Second, the characteristics of MDD patients might be a potential source of heterogeneity. Although we carried out the analysis under a random effect model, we did not perform quantitative calculation to measure the heterogeneity derived from patients' characteristics. Third, NMA required reasonably homogeneous trials. We did not adopt strict eligibility criteria for treatment duration and dose, and we combined studies with different antidepressants for the goal of larger number of included studies. Therefore, we might neglect the interaction between different anti-inflammatory agents and antidepressant agents. Finally, the numbers of included studies for all anti-inflammatory agents were relatively small, except for Omega-3 FA. Hence, the conclusions drawn from this NMA may be less robust and provide less power to guide clinical-decision making.

## Conclusions

In summary, we found that corticosteroids were more superior than other agents in terms of efficacy according to the SUCRA value. However, this result must be interpreted with caution because the head-to-head comparisons of the efficacy of glucocorticoids and other agents were not statistically significant. NSAIDs were recommended for acceptability and NAC for remission rate, but these findings should be interpreted cautiously due to some inevitable limitations. Therefore, more high-quality randomized clinical trials comparing different anti-inflammatory agents and investigating the optimal time, efficacy doses and intake duration are needed.

## Data Availability

The raw data supporting the conclusion of this article will be made available by the authors, without undue reservation.

## Supplementary Material

Supplementary Figure 1 Risk of bias graph.

Supplementary Figure 2 Risk of bias summary.

Supplementary Figure 3
**(A)** The forest plot of pairwise meta-analysis of efficacy. **(B)** The forest plot of pairwise meta-analysis of acceptability. **(C)** The forest plot of pairwise meta-analysis of remission. **(A-C)** legends: NSAIDs: non-steroidal anti-inflammatory drugs; NACs: N-acetylcysteines; Omega-3 FA: omega-3 fatty acid.

Supplementary Figure 4
**(A)** The PSRF of the model for efficacy. **(B)** The PSRF of the model for acceptability. **(C)** The PSRF of the model for remission. **(A-C)** legends: Approximate convergence is diagnosed when the upper limit was close to 1. 1: Placebo; 2: NSAIDs; 3: Corticosteroids; 4: Statins; 5: Pioglitazone; 6: Minocycline; 7: NACs; 8: Omega-3 FA.

Supplementary Figure 5
**(A1)** The density plot and trace plot of the model for efficacy. **(A2)** The density plot and trace plot of the model for efficacy. **(B1)** The density plot and trace plot of the model for acceptability. **(B2)** The density plot and trace plot of the model for acceptability. **(C1)** The density plot and trace plot of the model for remission. **(C2)** The density plot and trace plot of the model for remission. **(A-C)** legends: In the density plot, smooth density means sufficient accuracy. In the trace plot, the four different chains (dashed green, blue, red, and black lines) follow unclearly distinguishable paths, and their moving averages (solid lines) are identical between the first and second half of samples, which means sufficient accuracy. 1: Placebo; 2: NSAIDs; 3: Corticosteroids; 4: Statins; 5: Pioglitazone; 6: Minocycline; 7: NACs; 8: Omega-3 FA.

Supplementary Figure 6 Two-dimensional graphs about risk on efficacy and acceptability. Data are reported as RRs in comparison with placebo. Error bars are 95% CIs. Individual drugs are represented by different colored nodes.

Supplementary Figure 7
**(A)** Comparison-adjusted funnel plots of efficacy. **(B)** Comparison-adjusted funnel plots of acceptability. **(C)** Comparison-adjusted funnel plots of remission.

Supplementary Figure 8 1 Forest plots about adverse events of anti-inflammatory agents vs. placebo. 2 Forest plots about adverse events of anti-inflammatory agents vs. placebo. 3 Forest plots about adverse events of anti-inflammatory agents vs. placebo.
