# Supplementary figures and images for: Comparative Efficacy and Acceptability of Anti-inflammatory Agents on Major Depressive Disorder: A Network Meta-Analysis
Source: Front Pharmacol. 2021 Jul 1;12:691200. doi: 10.3389/fphar.2021.691200 (PMC8281269; doi:10.3389/fphar.2021.691200)

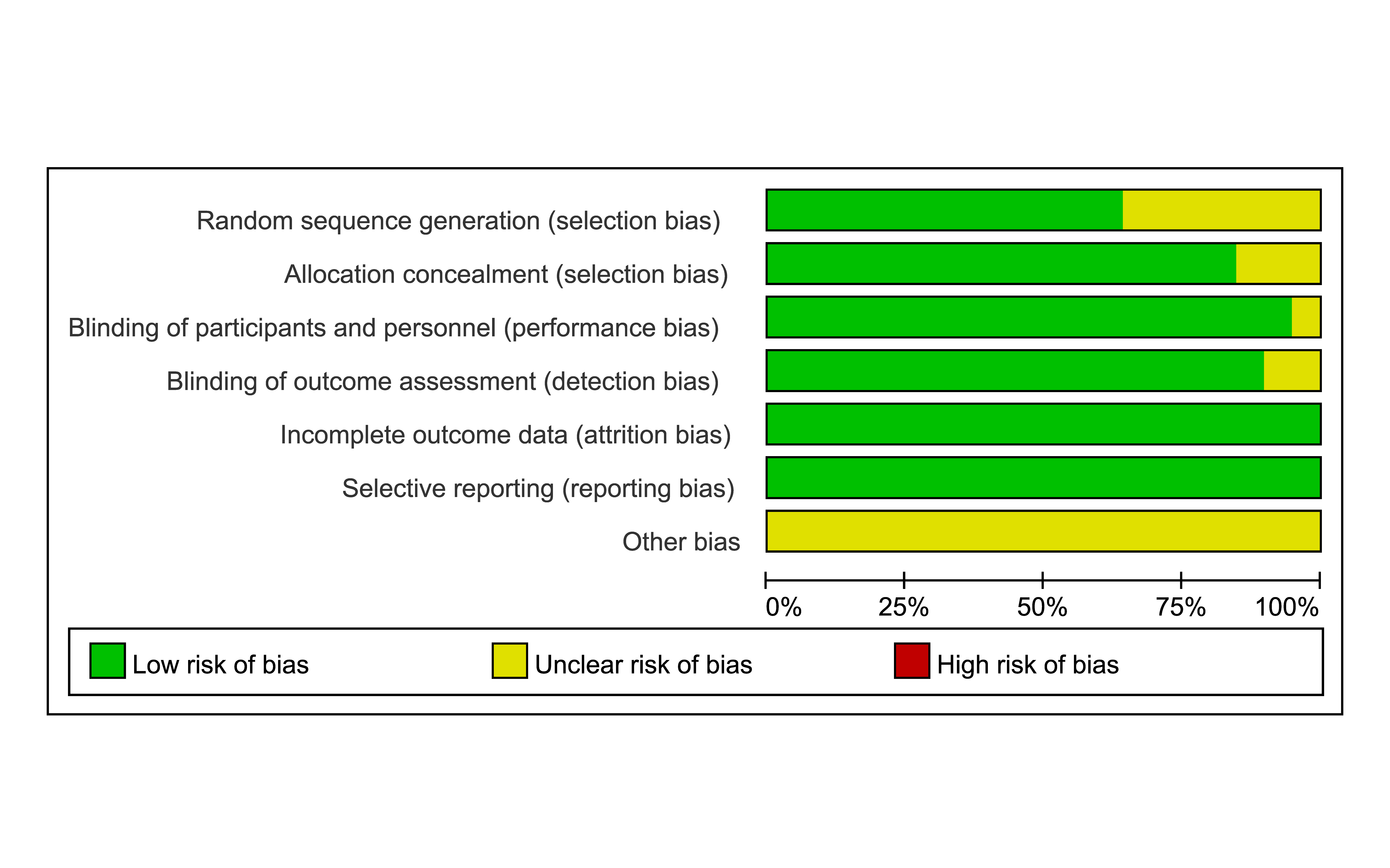

Supplement: Supplementary file 1 [file DataSheet1.ZIP › Figure S1 Risk of bias graph.tif]

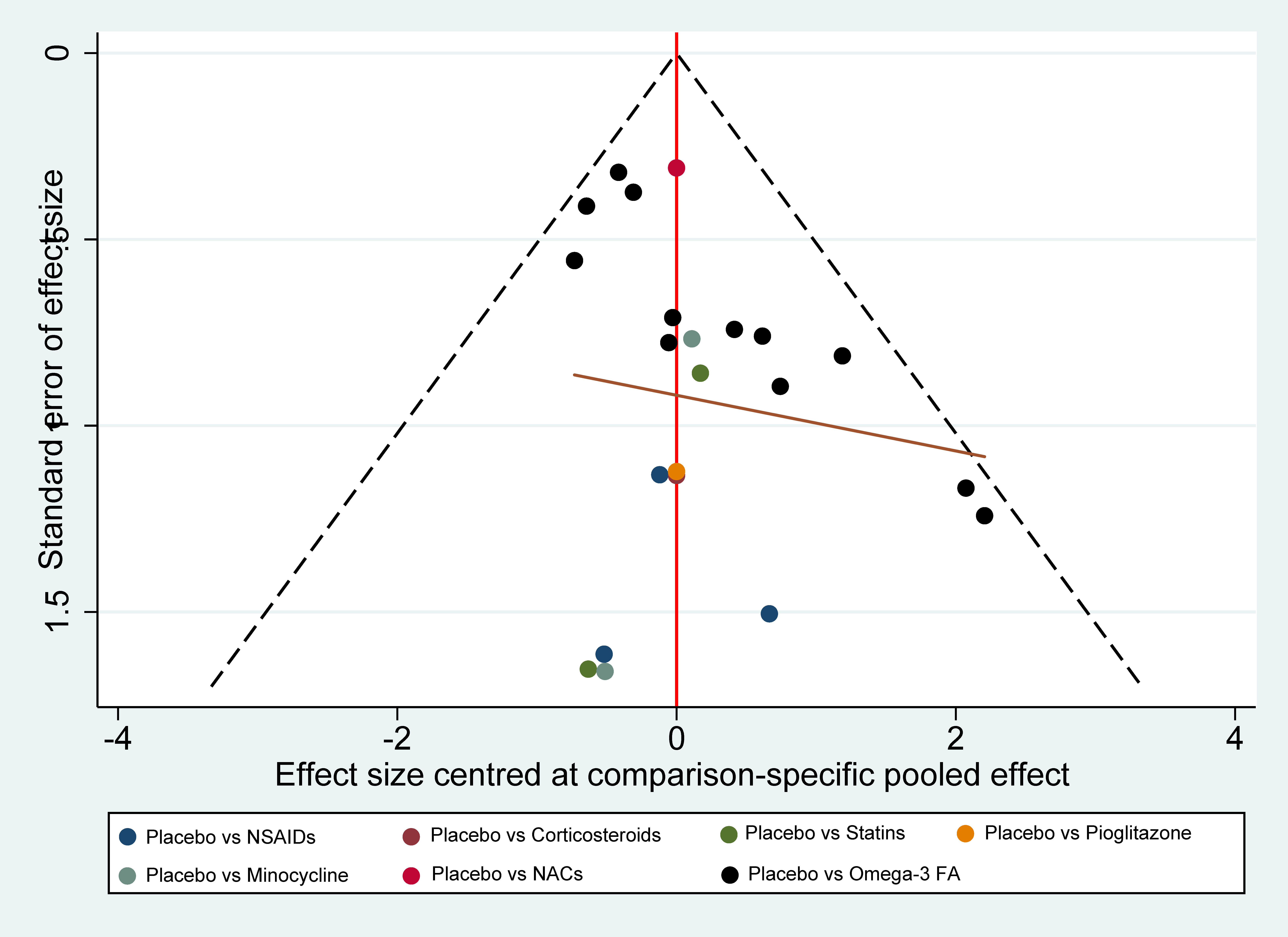

Supplement: Supplementary file 1 [file DataSheet1.ZIP › Figure S7 (A) Comparison-adjusted funnel plots of efficacy.tif]

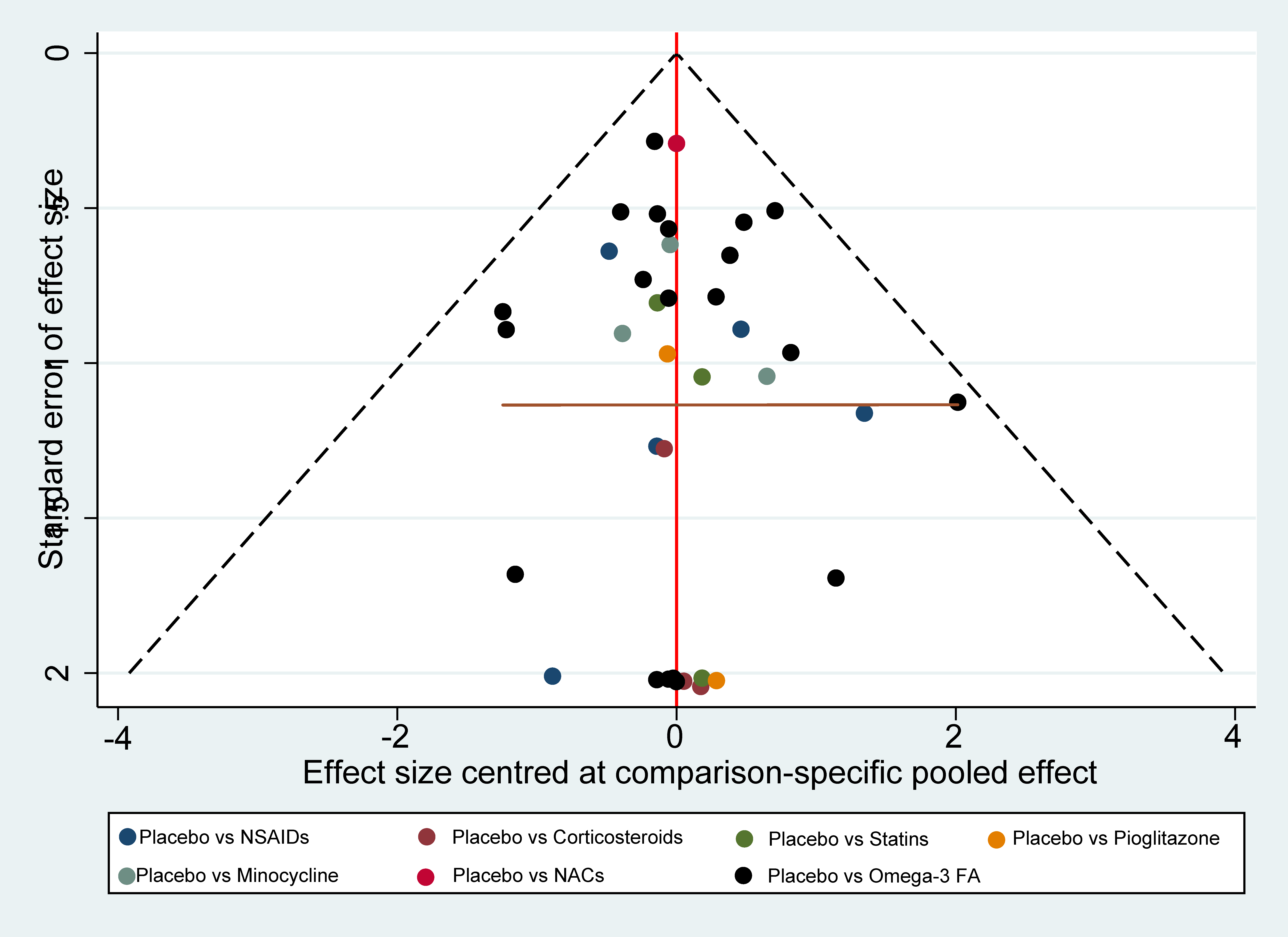

Supplement: Supplementary file 1 [file DataSheet1.ZIP › Figure S7 (B) Comparison-adjusted funnel plots of acceptability.tif]

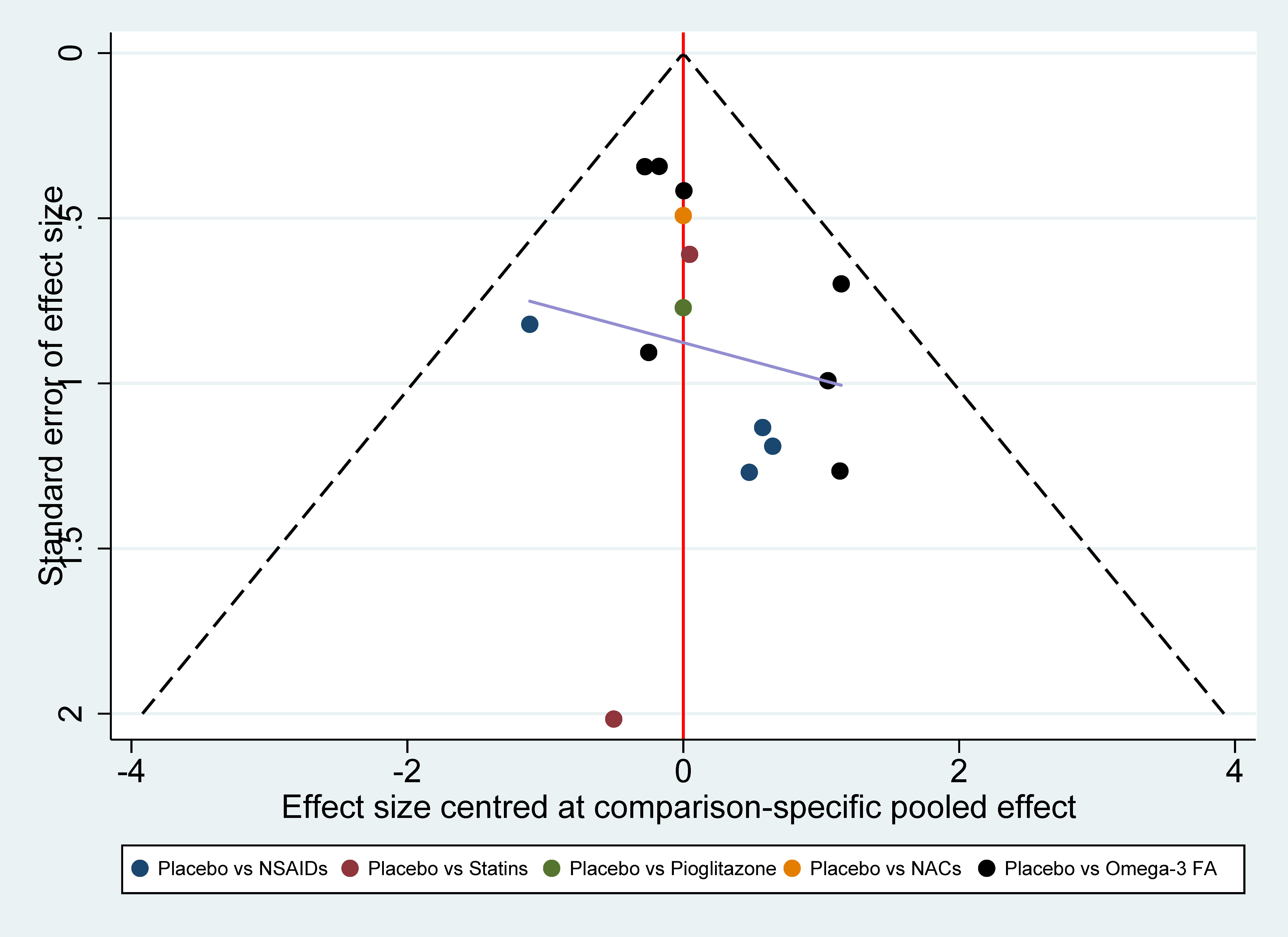

Supplement: Supplementary file 1 [file DataSheet1.ZIP › Figure S7 (C) Comparison-adjusted funnel plots of remission.tif]

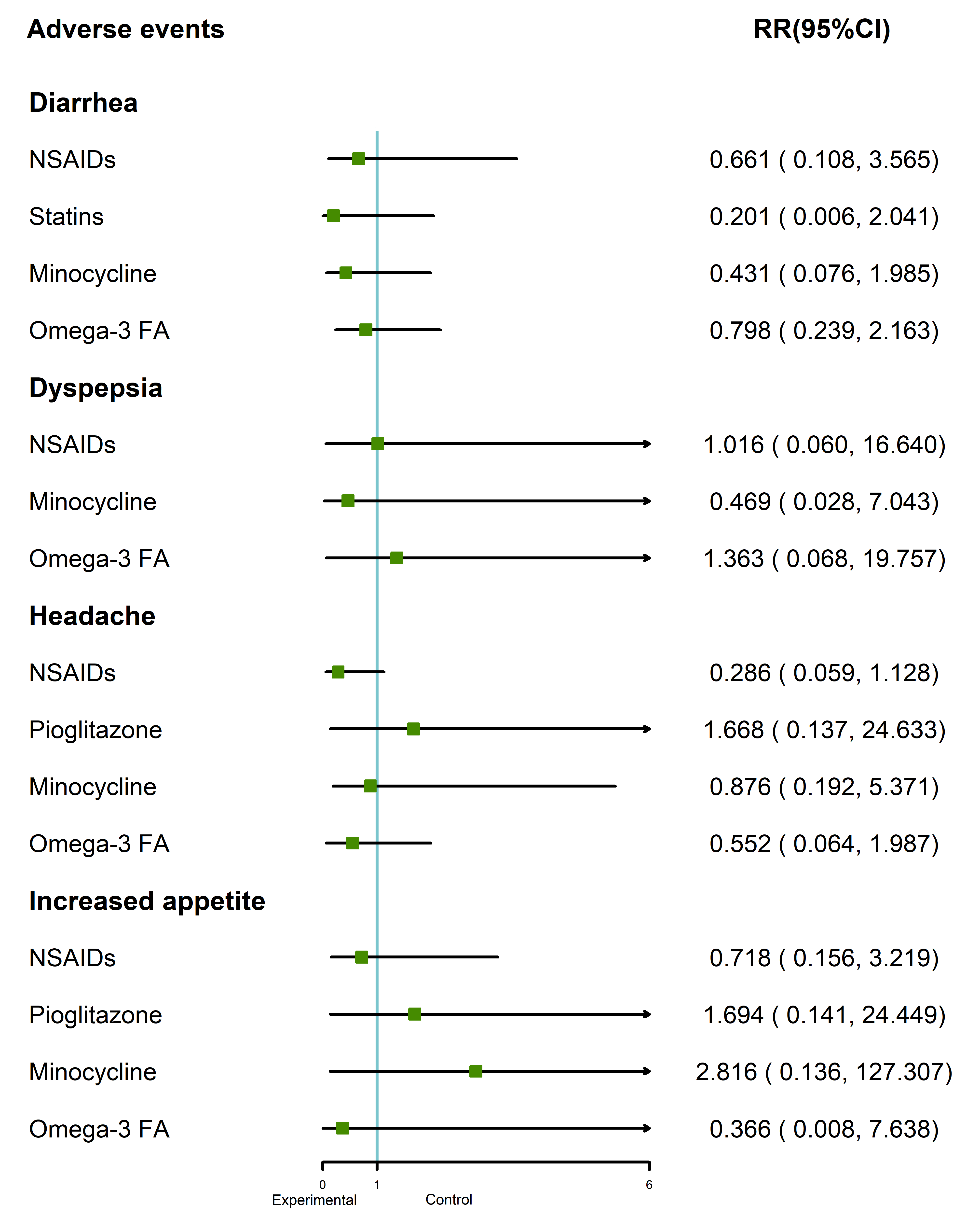

Supplement: Supplementary file 1 [file DataSheet1.ZIP › Figure S8-1 Forest plots about adverse events of anti-inflammatory agents vs. placebo.tif]

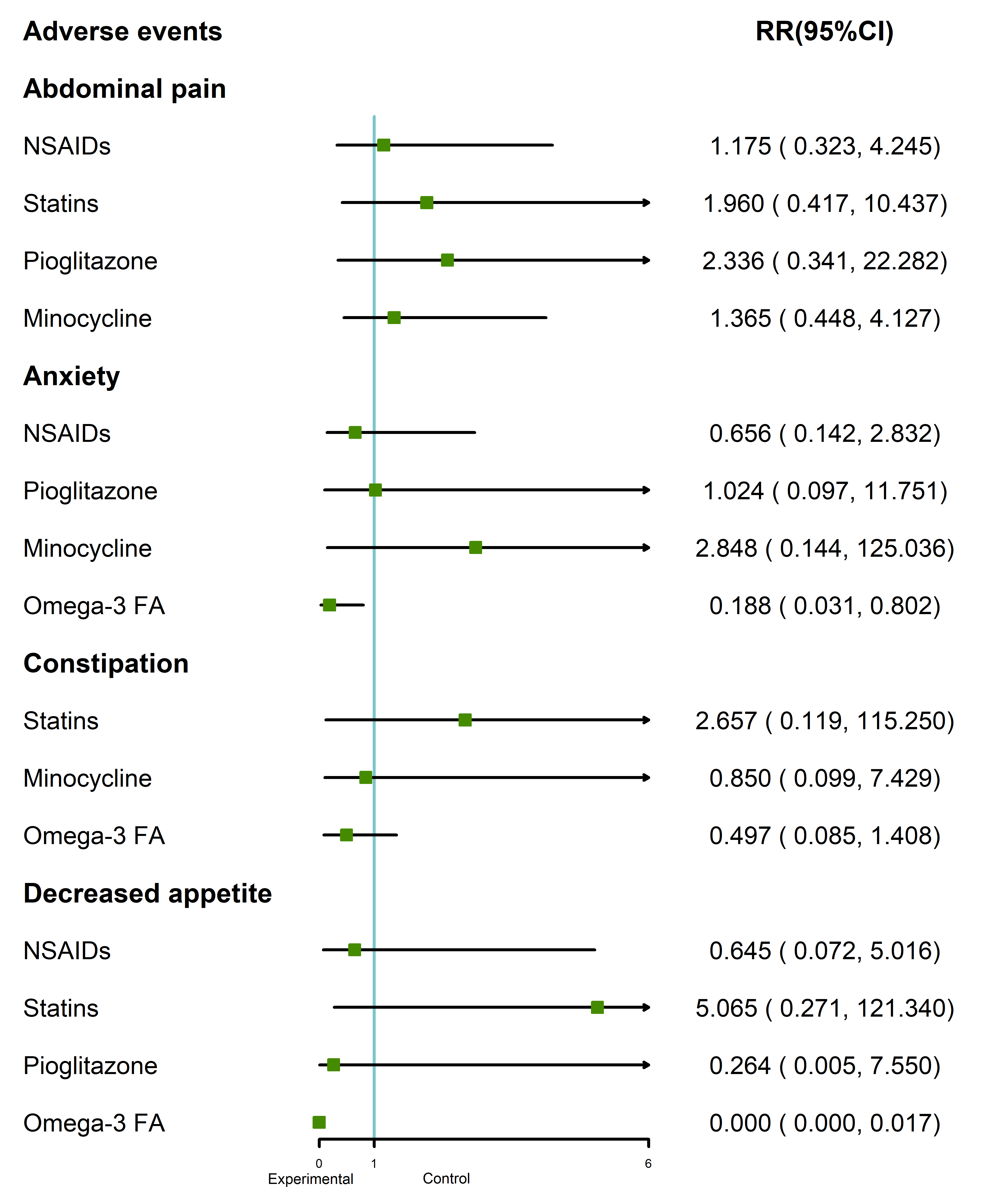

Supplement: Supplementary file 1 [file DataSheet1.ZIP › Figure S8-2 Forest plots about adverse events of anti-inflammatory agents vs. placebo.tiff]

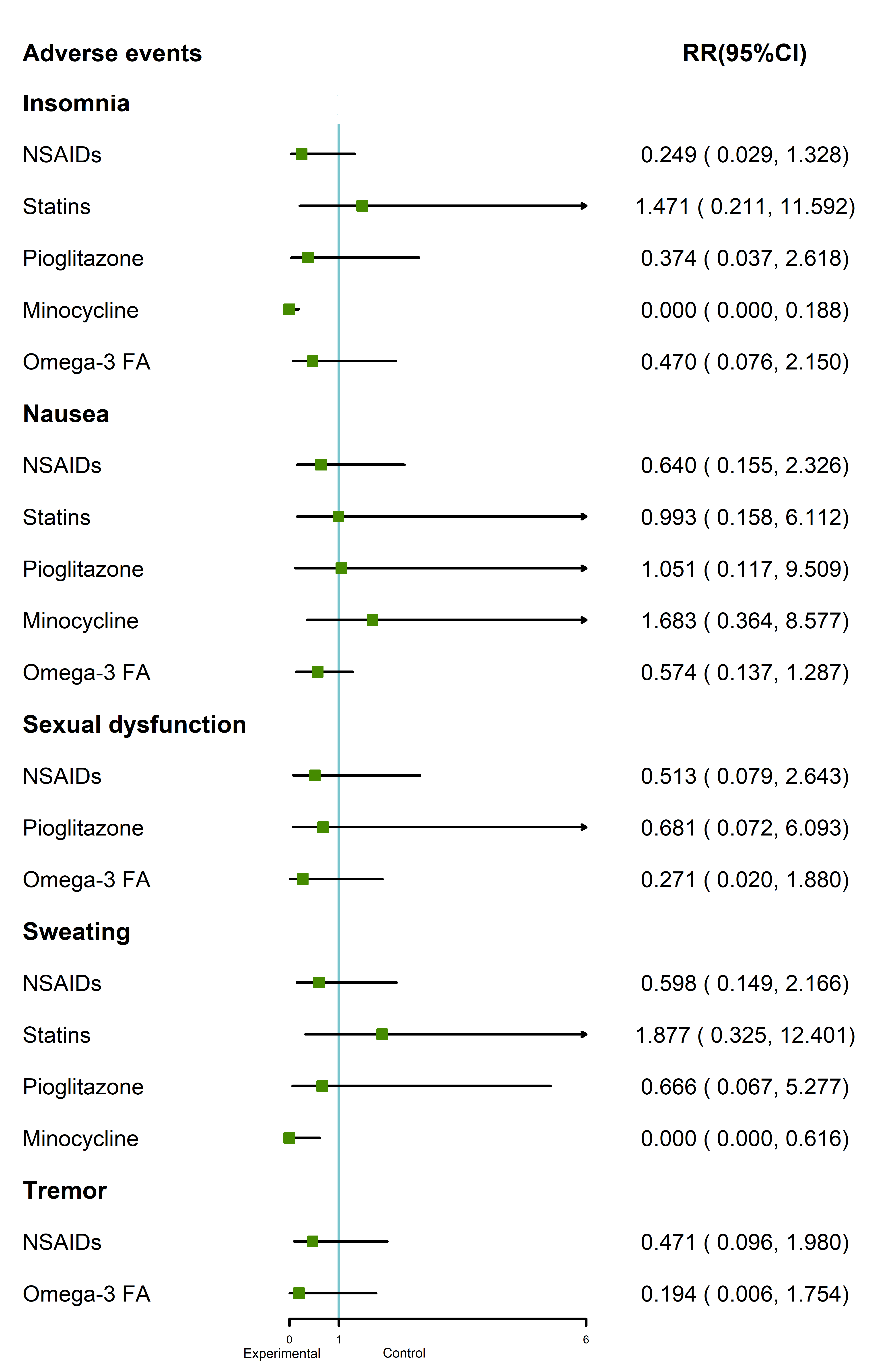

Supplement: Supplementary file 1 [file DataSheet1.ZIP › Figure S8-3 Forest plots about adverse events of anti-inflammatory agents vs. placebo.tif]
