# Supplementary material for: Comparative Efficacy and Acceptability of Anti-inflammatory Agents on Major Depressive Disorder: A Network Meta-Analysis
Source: Front Pharmacol. 2021 Jul 1;12:691200. doi: 10.3389/fphar.2021.691200 (PMC8281269; doi:10.3389/fphar.2021.691200)
Supplement: Supplementary file 2 [file DataSheet2.ZIP › Table S2 The residual deviance of outcome.docx]

Table S2 The residual deviance of outcome

| Outcome | Random model | Fixed model |
| --- | --- | --- |
| Efficacy | 46.7 | 50.3 |
| Acceptability | 75.9 | 77.5 |
| Remission | 32.3 | 33.1 |

Residual deviance represents the contribution of 1 data point for each study arm in a well-fitting model. The smaller the deviance, the better the fit.
