# Supplementary material for: Comparative Efficacy and Acceptability of Anti-inflammatory Agents on Major Depressive Disorder: A Network Meta-Analysis
Source: Front Pharmacol. 2021 Jul 1;12:691200. doi: 10.3389/fphar.2021.691200 (PMC8281269; doi:10.3389/fphar.2021.691200)
Supplement: Supplementary file 2 [file DataSheet2.ZIP › Table S3 The result of meta-regression .docx]

Table S3 The result of meta-regression

| Covariate | Median coefficient | 95%CI | Between-study  standard deviation | DIC |
| --- | --- | --- | --- | --- |
| **Efficacy** |  |  |  |  |
| No regression | - | - | 0.153 (0.011, 0.486) | 74.2 |
| Monotherapy or adjunctive therapy | -0.163 | (-0.718, 0.245) | 0.207 (0.015, 0.564) | 74.7 |
| Duration of treatment | -0.083 | (-0.726, 0.612) | 0.161 (0.009, 0.533) | 75.3 |
| Gender | 1.139 | (-0.584, 16.335) | 0.159 (0.010, 0.502) | 74.5 |
| **Acceptability** |  |  |  |  |
| No regression | - | - | 0.027 (0.001,0.093) | 108.5 |
| Monotherapy or adjunctive therapy | 0.015 | (-0.066,0.095) | 0.033 (0.002,0.104) | 109.7 |
| Duration of treatment | -0.006 | (-0.115,0.097) | 0.031 (0.001,0.101) | 109.7 |
| Gender | 0.129 | (-0.010,0.440) | 0.026 (0.001,0.088) | 106.6 |
| **Remission** |  |  |  |  |
| No regression | - | - | 0.156 (0.006,0.616) | 52.2 |
| Monotherapy or adjunctive therapy | 0.219 | (-0.418,0.934) | 0.179 (0.006,0.691) | 53.2 |
| Duration of treatment | -0.407 | (-1.429,0.505) | 0.170 (0.007,0.655) | 52.5 |
| Gender | 0.583 | (-0.241,1.497) | 0.134 (0.007,0.584) | 50.8 |

If 95%CI contain 0, it means covariate doesn't exhibit a significant coefficient in the interaction model.
